# Paraneoplastic syndrome – unresponsive systemic hypertension and fever as expression of primitive ciliary body carcinoma in a blind painful eye: a case report

**DOI:** 10.1186/s13256-015-0782-6

**Published:** 2016-01-18

**Authors:** Laura Balia, Paolo Vinciguerra, Alessandra Di Maria, Raffaele Piscopo, Mary Romano

**Affiliations:** 1 Department of Ophthalmology, Istituto Clinico Humanitas, IRCCS, Via Alessandro Manzoni 56, 20089 Rozzano, MI Italy; 2 Department of Ophthalmology, Second University of Neaples, Naples, Italy

**Keywords:** Cancer, Ciliary body carcinoma, Systemic hypertension

## Abstract

**Background:**

The purpose of this study was to present a case of paraneoplastic systemic hypertension and fever in an undiagnosed primary ciliary body carcinoma arising in a painful blind eye.

**Case presentation:**

A 37-year-old white man with a history of blindness since childhood was enucleated for neovascular glaucoma because of intractable pain in his right eye. A histopathological examination revealed a ciliary body carcinoma. One year later, an invasive recurrence of his orbit and ethmoid was detected and a debulking procedure was performed. He had untreatable fever and multidrug-resistant systemic hypertension for 3 months before the neoplasm diagnosis. He recovered from fever and systemic hypertension only after tumor excision and relapsed 1 year later when synchronous tumor dissemination was shown through a computed tomography scan. Tumor metastases, despite surgery and chemotherapy, caused his death.

**Conclusions:**

Paraneoplastic symptoms such as fever and hypertension may be due to unrecognized ocular malignancy. This case report intends to emphasize the importance of histopathological examination of an enucleated phthisical painful blind eye.

## Background

Alterations of visual function as a paraneoplastic manifestation of extraocular tumors have been described in the literature. Carcinoma and melanoma-associated retinopathy, cancer-associated cone dysfunction and bilateral uveal melanocytic proliferation are considered paraneoplastic syndromes due to circulating anti-retinal auto-antibodies and immune toxic factors released by the primitive tumors [1]. In this case report we describe a case of paraneoplastic symptoms associated with a primitive ciliary body carcinoma. Intractable fever and hypertension have not been reported as paraneoplastic signs related to ocular malignancy.

## Case presentation

**Fig. 1:**
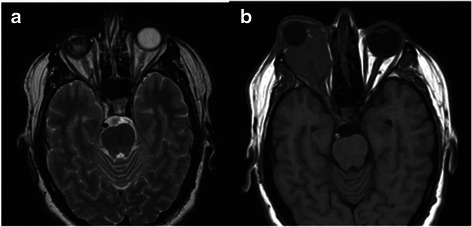
Magnetic resonance imaging scans showing **a** the atrophic right eye and **b** a large mass occupying the entire right socket displacing the prosthesis, reaching the optic foramen and eroding the bone wall of orbit and ethmoid

A 37-year-old white man came under our observation 8 years ago (2007) because of pain and increasing intraocular pressure despite maximal drug therapy in diagnosis of neovascular glaucoma in his right eye (RE). His medical history included blindness since childhood, polar posterior cataract and strabismus surgery in his RE at the age of 5 years. He has always had general good health, his left eye visual acuity was 20/20 and no ocular abnormality was observed. Magnetic resonance imaging (MRI) of his orbit and A-scan ultrasonography showed bulbar subatrophy, vitreous haemorrhage, and choroidal calcifications (Fig. 1a). He had systemic multidrug-resistant hypertension and a fever resistant to multiantibiotic therapy for 3 months before presentation.

**Fig. 2:**
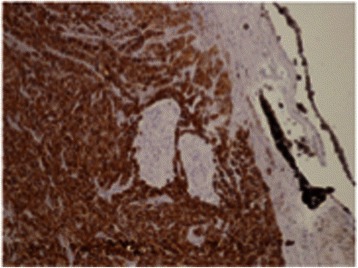
Tumoral specimen. Immunohistochemical staining for cytokeratin pool in the setting of solid proliferation of undifferentiated large cells with eosinophilic cytoplasm, atypical nuclei with prominent nucleoli and high mitotic activity with atypical figures

After 1 month he underwent enucleation to relieve the pain and a hydroxyapatite prosthesis, 18 mm in size, was implanted. A histopathological examination disclosed an undifferentiated carcinoma arising from the ciliary body. An immunohistochemical study was positive for cytokeratin pool and negative for S-100, HMB45, Melan-a, CD34, CD45, CD3, and CD20 (Fig. 2). A total body computed tomography (CT) scan, head and neck MRI were performed and no signs of metastatic disease were detected. A longitudinal follow-up was carried out revealing no remarkable signs. Since June 2008 (1 year after the enucleation procedure) he again had severe head and neck pain, fever and systemic hypertension. A new CT scan and MRI demonstrated a mass involving his entire orbital cavity displacing the prosthesis, which reached his optic foramen and medially eroded the bone wall of his orbit and ethmoid (Fig. 1b). He underwent a debulking procedure followed by three chemotherapy cycles (cisplatin and 5-fluorouracil). After the surgery his blood pressure improved significantly and remained controlled with single therapy and without fever. In September 2008 a new head and neck CT scan and MRI showed a relapse. We decided to perform a resection of the tumor involving front and middle skull base and his fronto-orbito-ethmoidal area. The following year a CT total body scan showed bone, lung, adrenal, and retroperitoneal metastases and he died 5 years ago, despite radiotherapy and six chemotherapy cycles: Taxotere (docetaxel), cisplatin and 5-fluorouracil.

## Discussion

Paraneoplastic syndromes may be the first signs of malignancy. They are defined as systemic tumor-associated signs and symptoms not directly related to the site of a malignant tumor or its metastases. The symptoms may be endocrine, neuromuscular or musculoskeletal, cardiovascular, coetaneous, hematologic, gastrointestinal, renal, ocular, or miscellaneous. These phenomena are mediated by humoral factors (hormones or cytokines) excreted by tumor cells or by an immune response against the tumor and they could occur in a variety of malignant neoplasms [2].

Paraneoplastic clinical signs are not directly related to the site or local manifestations of a malignant tumor or its metastases. Ocular paraneoplastic syndromes have been described as being due to circulating anti-retinal auto-antibodies and immune toxic factors released by the extraocular primitive tumors. Carcinoma-associated retinopathy, melanoma-associated retinopathy, cancer-associated cone dysfunction and bilateral uveal melanocytic proliferation are considered paraneoplastic syndromes [1].

We report a case of systemic hypertension and fever as paraneoplastic manifestations in a patient affected by primary body ciliary neoplasm.

Rare paraneoplastic syndromes, which can produce systemic hypertension, may be associated with hepatocarcinoma or renal malignancies, while it is usually alleged that more than half of patients with cancer will experience fever during the course of their disease [3]. The reasons for cancer-related fever are multiple but the most frequent and severe causes include opportunistic infections, venous thromboembolic disease or, more rarely, the fever is tumor-treatment related [4].

To the best of our knowledge, paraneoplastic untreatable fever and systemic hypertension have not been related to ocular malignancy.

Moreover, previous accurate examinations and radiological imaging failed to show an orbital mass whereas a histopathological examination revealed a primitive ciliary body carcinoma.

These tumors arising from the pigment epithelium of the ciliary body are exceptionally rare and benign, but their extension beyond the globe leads to a worse prognosis. It has been previously reported that in phthisical eyes, with or without a history of trauma or inflammation, a malignant transformation may occur probably due to reactive hyperplasia of the ciliary epithelium [5].

## Conclusions

In this case we did not consider our patient’s antibiotic-resistant fever and intractable systemic hypertension to be paraneoplastic syndrome which resolved after total surgical removal of the primitive tumor and relapsed with neoplastic recurrences.

Physicians should be aware of systemic symptoms such as systemic hypertension unresponsive to drugs and fever in phthisic eye: they may be paraneoplastic signs of ocular malignancy because they are related to paraneoplastic or tumor necrosis.

Furthermore, in the presence of a painful blind eye the differential diagnosis between neovascular glaucoma and any form of bulbar tumor has to be considered and histopathological examination should always be performed in order to prevent any malignant dissemination.

## Consent

Written informed consent was obtained from the patient’s next-of-kin for publication of this case report and any accompanying images. A copy of written consent is available for review by the Editor-in- Chief of this journal.

## Abbreviations

CT: computed tomography
MRI: magnetic resonance imaging
RE: right eye
